# Residual renal volume as a long-term independent predictive factor of developing chronic kidney disease after donor nephrectomy

**DOI:** 10.1038/s41598-024-55499-3

**Published:** 2024-03-04

**Authors:** Thanakhom Hoontrakul, Charoen Leenanupunth, Mookdarat Siantong, Pokket Sirisreetreerux, Sith Phongkitkarun, Wisoot Kongchareonsombat, Kittinut Kijvikai

**Affiliations:** 1 Faculty of Medicine, Thammasat University, Pathumthani, Thailand; 2 Division of Urology, Department of Surgery, Faculty of Medicine Ramathibodi Hospital, Mahidol University, Bangkok, Thailand; 3 Department of Radiology, Faculty of Medicine Ramathibodi Hospital, Mahidol University, Bangkok, Thailand

**Keywords:** Donor nephrectomy, Chronic kidney disease, Residual renal volume, Long-term follow-up, Renal replacement therapy, Nephrology, Urology

## Abstract

To assess the long-term association between the residual renal volume and the progression of chronic kidney disease (CKD) in kidney donors following open or laparoscopic donor nephrectomy. A retrospective observational study involving 452 individuals who underwent open or laparoscopic donor nephrectomy at Ramathibodi Hospital, Bangkok, Thailand. The study spanned over a comprehensive 60-month monitoring period. Residual renal volume was determined through Computer Tomography. Patient characteristics, surgical techniques, donated kidney side, and estimated glomerular filtration rate (eGFR) were collected and analysed. In a multivariate analysis, a residual renal volume exceeding 50% of original volume is associated with an increased likelihood of developing CKD, with a hazard ratio (HR) of 1.675 (P < 0.05), and male gender has a hazard ratio (HR) of 4.013 (P < 0.001). Additionally, age is identified as a minor risk factor for developing CKD, with hazard ratio (HR) of 1.107 (P < 0.001). Higher residual renal volume, male gender, and older age were identified as independent risk factors for the development of CKD following open or laparoscopic donor nephrectomy during long-term follow-up.

## Introduction

Chronic kidney disease (CKD) represents a significant global public health challenge, with a reported global prevalence of 13.4%, and end-stage renal disease (ESRD) affecting approximately 5–7 million individuals worldwide^1^. In Thailand, CKD affects over 11 million individuals, which accounts for 17.5% of the population, and 5.7 million people, or 8.6%, require renal replacement therapy for ESRD^2^.

Kidney transplantation (KT) is the primary standard for renal replacement therapy in cases of end-stage renal disease (ESRD)^3^. Research has consistently shown that patients who undergo KT experience better outcomes, including lower mortality rates, improved quality of life, and cost-effectiveness, when compared to those receiving haemodialysis^4,5^. Moreover, it is well-established that kidneys from living donors offer higher graft survival rates and lower mortality rates than kidneys from deceased donors^6,7^. In 2020, Thailand recorded a total of 714 kidney transplantations, with 136 involving living donors and 578 from deceased donors^8,9^.

Research has indicated that both open donor nephrectomy and laparoscopic donor nephrectomy yield comparable graft outcomes^10,11^. However, it is worth noting that donor nephrectomy carries a slight risk of inducing chronic kidney disease (CKD) and end-stage renal disease (ESRD) in the donor, estimated to be around 1–5%^12,13^. A previous study discovered that donors with a pre-donation renal volume of less than 140 ml face an increased risk of developing CKD within 12 months^14^. Similarly, another study suggested that preoperative renal volume serves as a protective factor against CKD, although this finding lacks long-term follow-up^15^. Consequently, our current study aims to assess the correlation between residual kidney volume and the risk of CKD following donor nephrectomy, with an extended follow-up period.

## Methodology

### Study population

This research received ethical approval from the Institutional Committee on Human Rights Related to Research Involving Human Subjects (MURA2019/855). All methods were carried out in accordance with relevant guidelines and regulations. A retrospective observational study was conducted on all patients who underwent open and laparoscopic donor nephrectomy between January 2011 and June 2019. The data collection was retrospective in nature and informed consent was waived by the Institutional Committee on Human Rights Related to Research Involving Human Subjects. In Thailand, a potential kidney donor should possess an estimated glomerular filtration rate (eGFR) exceeding 90 ml/min/1.73 m^2^. Individuals with a measured glomerular filtration rate (mGFR) below 70 ml/min/1.73 m^2^ are not considered eligible to be kidney donors. Additionally, they undergo assessments for proteinuria, haematuria, malignancies, and metabolic conditions that could impact both the transplantation outcomes and their post-procedural health. Screening for infectious and psychological diseases is also conducted. Furthermore, a CT angiography is essential for a thorough anatomical evaluation. Data collection extended until the last follow-up visit. Information collected included age, gender, body weight, height, body mass index (BMI), donated kidney side, residual renal volume, underlying diseases including diabetes mellitus, hypertension, dyslipidaemia, hepatitis B viral infection and estimated Glomerular Filtration Rate (eGFR). eGFR values were calculated using the Chronic Kidney Disease Epidemiology Collaboration (CKD-EPI) method^16^, with CKD criteria set at eGFR < 60 ml/min/1.73 m^2^ for two consecutive follow-ups. Preoperative computed tomography (CT) scans were routinely utilized to assess renal volume. Patients were excluded if they lacked follow-up records and eGFR results beyond one month, or if their imaging data was incomplete.

### Imaging procedure

Extended Brilliance Workspace system software, version 4.5.6.52040 (Philips Medical Systems, Markham, Ontario, Canada) were used to perform Computed Tomography Angiography (CTA) in order to formulate the preoperative renal volume for all patients. The images were oriented in the transverse plane to manually delineate the contours of the kidneys. We excluded the drainage system, vasculature of the renal sinus, adipose tissues in the renal sinus, and any cysts. The remaining renal volume was automatically computed using post-processing software. Furthermore, a technician conducted the measurements independently, performing two separate repetitions, without prior knowledge of the participants' characteristics.

### Statistical analysis

Patients' baseline characteristics were reported in mean ± standard deviation (SD) or median with interquartile range (IQR). Univariate and multivariate analyses with cox proportional regression models were used to analyse the factors affecting long term kidney function. Correlation analyses were drawn for residual renal volume and CKD. All the data were analysed using Stata software, version 14.2 (StataCorp LLC, College Station, TX, USA).

## Results

The study comprised 452 patients who had undergone donor nephrectomy. Among these, 289 individuals (63.94%) were female, and 163 (36.06%) were male. The average age of the donors was 39.57 ± 11.00 years (mean ± SD), as indicated in Table 1. A majority of the patients underwent donor nephrectomy on the left side (81.42%). The surgical procedures included open nephrectomy for 188 donors and laparoscopic nephrectomy for 264 donors. Twenty-three patients (5.09%), had underlying conditions such as dyslipidaemia, hypertension, or hepatitis B viral infection.

**Table 1:** Characteristic data by side of donated kidney.

Variable	Total (n = 452)	Right (n = 84)	Left (n = 368)	p-value
Gender, n (%)				
Female	289 (63.94)	49 (58.33)	240 (65.22)	0.236
Male	163 (36.06)	35 (41.67)	128 (34.78)	
Underlying disease, n (%)				
No	429 (94.91)	78 (92.86)	351 (95.36)	0.342
Yes	23 (5.09)	6 (7.14)	17 (4.62)	
Age(year), mean ± SD	39.57 ± 11.00	40.03 ± 11.27	39.47 ± 10.95	0.672
Surgical approach, n (%)				
Open	188 (41.59)	78 (92.86)	110 (29.89)	< 0.001
Laparoscopic	264 (57.41)	6 (7.14)	258 (70.11)	
GFR (ml/min/1.73 m^2^), mean ± SD	106.26 ± 21.62	106.65 ± 12.32	106.17 ± 23.23	0.856
BMI, mean ± SD	24.33 ± 3.90	25.00 ± 4.49	24.18 ± 3.74	0.080
Renal volume (ml), mean ± SD				
Total	252.40 ± 46.64	260.32 ± 46.95	250.60 ± 46.44	0.084
% remain	50.59 ± 2.34	49.53 ± 2.56	50.83 ± 2.22	< 0.001

Total = total renal volume (ml).

% remain = percentage of residual renal volume.

Prior to the surgery, the average total renal volume was 252.40 ± 46.64 ml. Patients who underwent right nephrectomy had a higher renal volume compared to those who had left nephrectomy, as outlined in Table 1. It is worth noting that a statistically significant difference exists between the percentage of remaining renal volume on the right side (128.87 ± 23.88 ml, 49.53 ± 2.56%) and the left side (127.40 ± 24.23 ml, 50.83 ± 2.22%), with a p-value of less than 0.001. In terms of remaining renal volume, gender, age, and underlying diseases did not exhibit significant differences between the two groups, as shown in Table 2.

**Table 2:** Characteristic data by remaining renal volume.

Variable	Total (n = 452)	Remain volume < 50% (n = 159)	Remain volume > 50% (n = 293)	p-value
Gender, n (%)				
Female	289 (63.94)	103 (64.78)	186 (63.48)	0.784
Male	163 (36.06)	56 (35.22)	107 (36.52)	
Underlying disease, n (%)				
No	429 (94.91)	151 (94.97)	278 (94.88)	0.968
Yes	23 (5.09)	8 (50.3)	15 (5.12)	
Age (year), mean ± SD	39.57 ± 11.01	38.93 ± 10.47	39.93 ± 11.28	0.358
Operation type, n (%)				
Open	84 (15.58)	52 (32.70)	32 (10.92)	< 0.001
Laparoscope	368 (81.42)	107 (67.30)	261 (89.08)	
Side, n (%)				
Right	188 (41.59)	85 (53.46)	103 (35.15)	< 0.001
Left	264 (58.41)	74 (46.54)	190 (64.85)	
GFR (ml/min/1.73 m^2^), mean ± SD	106.26 ± 21.62	105.69 ± 13.55	106.56 ± 24.88	0.690
BMI, mean ± SD	24.33 ± 3.90	24.61 ± 3.99	24.17 ± 3.85	0.250
Renal volume (%), mean ± SD				
Total	252.41 ± 46.64	255.85 ± 50.74	250 ± 44.23	0.267
%loss	49.41 ± 2.34	51.59 ± 1.65	48.22 ± 1.72	< 0.001

Total = total renal volume (ml).

%loss = percentage of renal volume loss from donor nephrectomy.

Table 3 illustrates that 33.09% of donors developed eGFR deterioration within 1 month after nephrectomy and eGFR gradually decreased over the 24 months follow-up period with some minor variations. After the 24 months follow-up, the proportion of donors who developed CKD remained uniform at approximately 20%. The data lacks information on 41 patients who were lost to follow-up after the first month.

**Table 3:** CKD within 60 month (n = 411).

Variable	Total (n = 411)	Non CKD (GFR ≥ 60) ml/min/1.73 m^2^	CKD (GFR < 60) ml/min/1.73 m^2^
Follow-up time			
1 month	411 (100)	275 (66.91)	136 (33.09)
3 month	256 (100)	183 (71.48)	73 (28.52)
6 month	275 (100)	195 (70.91)	80 (29.09)
12 month	308 (100)	234 (75.97)	74 (24.03)
18 month	310 (100)	236 (76.13)	74 (23.87)
24 month	170 (100)	123 (72.35)	47 (27.65)
30 month	210 (100)	168 (80.00)	42 (20.00)
36 month	201 (100)	162 (80.60)	39 (19.40)
42 month	193 (100)	157 (81.35)	36 (18.65)
48 month	170 (100)	136 (80.00)	34 (20.00)
54 month	166 (100)	139 (83.73)	27 (16.27)
60 month	129 (100)	105 (81.40)	24 (18.60)

Remarks: GFR (ml/min/1.73 m^2^) of donors may fluctuate between CKD and non-CKD, meaning that the same donor may meet the CKD criteria more than once.

In reference to the risk factors associated with postoperative chronic kidney disease (CKD), the statistical analysis highlights that being male, advancing age, and the remaining kidney volume are significant factors. The hazard ratios (HR) with 95% confidence intervals (CI) are 4.013 (2.67- 6.03) for male gender, 1.107 (1.08–1.13) for age, and 1.675 (1.09–2.58) for remaining volume, as presented in Table 4.

**Table 4:** Risk factor to CKD.

Variable	Univariate		Multivariate	
	HR (95%CI)	p-value	HR (95%CI)	p-value
Gender				
Female	1		1	
Male	2.65 (1.81–3.85)	< 0.001	4.01 (2.67–6.03)	< 0.001
Age(year)	1.10 (1.07–1.12)	< 0.001	1.11 (1.08–1.13)	< 0.001
BMI	1.10 (1.02–1.12)	0.007	1.05 (0.99–1.11)	0.053
Operation type				
Open	1			
Laparoscope	1.53 (1.03–2.27)	0.035		
Renal volume (ml)				
% remain				
< 50%	1		1	
> 50%	1.57 (1.05–2.36)	0.028	1.68 (1.09–2.58)	0.019

% remain = percentage of residual renal volume.

**Figure 1:**
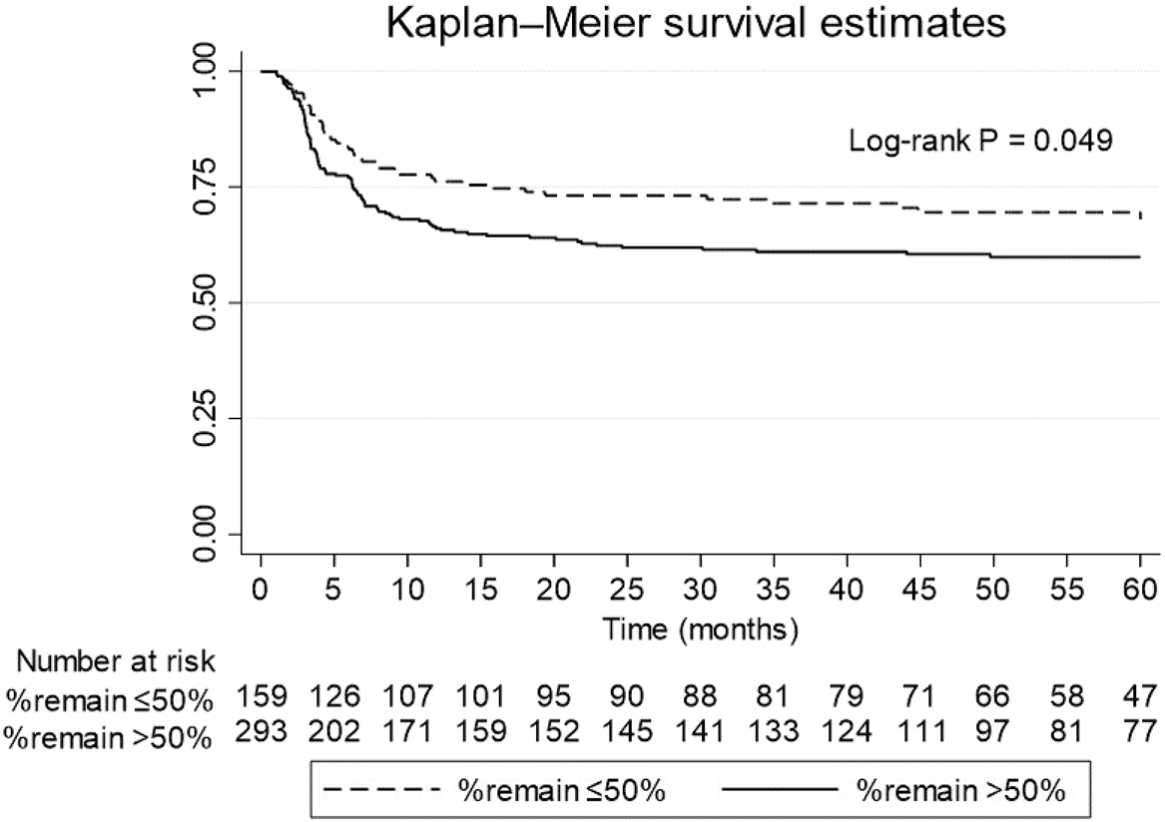
CKD development according to remnant kidney volume (> 50 or < 50%). Remark: the Y axis shows cumulative incidence of CKD development over time.

The Kaplan–Meier curve in Fig. 1 illustrates CKD development over a 5-year follow-up, comparing individuals with kidney remnants below 50% to those with remnants exceeding 50%. Those with greater kidney remnants exhibited a faster onset of CKD compared to those with less, with a Log rank P value of 0.049.

## Discussion

In our research, we examined the long-term renal function in kidney donors. We identified that gender, age, and greater remaining renal volume independently contribute to the risk of developing chronic kidney disease (CKD) following both open and laparoscopic donor nephrectomy procedures. Our results contradicted those reported in previous studies with differing follow-up durations^17,18^. Hori et al. and Lange et al. both proposed a correlation between kidney volume and kidney function before and after donation. Additionally, Narasimhamurthy et al. indicated that kidney size is linked to the long-term eGFR. In fact, one study has indicated a robust correlation between the volume of the renal cortex and eGFR after kidney donation^19^. The total parenchymal renal volume contouring (ReRCoV) method has demonstrated the most substantial predictive efficacy for the development of chronic kidney disease (CKD) one year after kidney donation, as per previous research^20^.

Our results indicate that higher residual renal volume is associated with developing CKD in long-term follow-up, which contradicted previous studies. Meta-analysis by Habbous et al. might help clarify the inconsistencies, as they discovered that the apparent link between renal volume and post-donation renal function was notably diminished due to factors such as unadjusted measures, risk stratification, and reliance on input distribution^21^.

Another factor contributing to the disparity between our findings and previous research could be the differences in the measurement and eGFR calculation methods. Moreover, there is a lack of standardized approach for measuring renal volume using CT scans, and the methods differ in terms of software, equipment, and procedures. These include variations such as ReRCoV, total parenchymal three-dimensional renal volume, renal cortical volume, kidney function cortical volumetry (RCoV), and the ellipsoid method^19,22^. According to a study, it was claimed that RCoV is the most effective volumetric method for predicting post-renal outcomes^19^. Furthermore, the absence of standardization in the quality of the arterial phase for corticomedullary differentiation of the kidney hinders the comparability of findings from other studies.

Regarding the calculation of eGFR, our study employed the CKD-EPI method, while another research used the four-variable Modification of Diet in Renal Disease (MDRD) equation. Additionally, a separate study compared the accuracy of three eGFR calculation methods but found them to be less reliable than the Chromium-51 labelled ethylenediaminetetraacetic acid radioisotope GFR^23^. Notably, in the context of evaluating kidney donors, CKD-EPI has demonstrated superior performance in eGFR measurement compared to MDRD, underscoring the absence of standardization in GFR measurement^24^. At our institution, the majority of surgical procedures were conducted using laparoscopic technique, with a preference for left kidney donation. This preference is primarily due to the left laparoscopic donor nephrectomy being less surgically intricate, owing to the anatomically longer renal vein^25,26^, facilitating vascular anastomoses. When dealing with kidneys featuring multiple renal arteries, we evaluate the length of vessels on a case-by-case basis. We opt for the side with less complex vasculature to mitigate the risk of post-transplant ischemia in the renal units. In cases of differing kidney functions, nuclear renal scintigraphy was assessed and the lower functioning kidney is chosen for transplantation. Donors with kidney stones are advised to have them removed before transplantation. Removal of small, uncomplicated renal stones in the chosen kidney can be done after nephrectomy promptly with endoscopy on the operating table to minimize ischemic time before proceeding with transplantation.

Our findings suggested that the residual renal volume is typically higher in left donor nephrectomies, implying that, in general, the right kidney is larger than the left, as supported by our data and previous studies^27^. This could potentially be a confounding factor influencing the results. Moreover, we identified male gender as a significant risk factor for chronic kidney disease (CKD). While numerous studies have reported similar findings, the underlying reasons for the connection between male gender and CKD in donor nephrectomy cases remain unclear^20,28^. Some studies have proposed that both age and male gender could impact the renal reserve capacity^29,30^. Additionally, a separate research study established a link between the cortex-aortic enhancement index and the decline in eGFR, particularly in relation to male donors^28^.

Our study has certain limitations. Primarily, it is a retrospective observational study, thus could have been influenced by incomplete data and potential biases. Additionally, there is a significant number of participants who were lost to follow-up, impacting the analysis due to the limited number of reported CKD cases. Finally, some participants who marginally met the criteria for CKD and subsequently showed improvement in the subsequent visit, only to experience a decline in eGFR, leading to them meeting the CKD criteria again. These cases essentially represent two distinct occurrences of CKD.

## Conclusion

Higher residual renal volume, increasing age and male gender were found to be independent risk predictors of developing CKD after open or laparoscopic donor nephrectomy in long-term follow-up.

## Further research

To facilitate cross-study comparisons, it is imperative to standardize measurements by assessing various imaging procedures, methods for measuring renal volume, and eGFR calculation methods. This standardization is crucial to identify the optimal approach for evaluating kidney donors. Subsequently, further research is warranted to elucidate the underlying mechanisms contributing to the increased susceptibility of males to developing CKD following donor nephrectomy.

## Data Availability

Data supporting this study are included within the article.
