# Author Correction: Residual renal volume as a long-term independent predictive factor of developing chronic kidney disease after donor nephrectomy

**DOI:** 10.1038/s41598-024-60551-3

**Published:** 2024-04-30

**Authors:** Thanakhom Hoontrakul, Charoen Leenanupunth, Mookdarat Siantong, Pokket Sirisreetreerux, Sith Phongkitkarun, Wisoot Kongchareonsombat, Kittinut Kijvikai

**Affiliations:** 1 Faculty of Medicine, Thammasat University, Pathumthani, Thailand; 2 Division of Urology, Department of Surgery, Faculty of Medicine Ramathibodi Hospital, Mahidol University, Bangkok, Thailand; 3 Department of Radiology, Faculty of Medicine Ramathibodi Hospital, Mahidol University, Bangkok, Thailand

Correction to: *Scientific Reports* 10.1038/s41598-024-55499-3, published online 04 March 2024

The original version of this Article contained an error in the name of Sith Phongkitkarun, which was incorrectly given as Sith Phongkitkarn.

The original Article has been corrected.

